# Demystification and feasibility of CRISPR technology and gene editing in African laboratories

**DOI:** 10.1242/bio.060122

**Published:** 2023-10-19

**Authors:** Mohamed Jemaà

**Affiliations:** ^1^ Human Genetics Laboratory, Faculty of Medicine of Tunis, Tunis El Manar University, Tunis 1006, Tunisia; ^2^ Department of Biology, Faculty of Science of Tunis, Tunis El Manar University, Tunis 2092, Tunisia; ^3^ Young Tunisian Researchers in Biology (YTRB) Network, Sfax 3000, Tunisia

**Keywords:** CRISPR, Gene editing, Low-budget

## Abstract

Clustered regularly interspaced short palindromic repeats, or CRISPR, is a powerful molecular biology tool that is enabling high-quality genetic research and engineering. However, for practical reasons, but more specifically because of the lack of training and the rapid development of gene-editing technology, the technique is still not well established in African laboratories. For this reason, a consortium formed by the Institut Pasteur of Tunis and Learn and Win decided to organise an international conference and workshop on CRISPR technology in particular and gene editing in general, focusing on the low-budget model more appropriate to the African context. From 12 to 17 June 2023, more than 200 interdisciplinary researchers and students from the life sciences and more than 20 international speakers and trainers gathered at the Institut Pasteur in Tunis, Tunisia, for the First African Conference and Workshop on CRISPR to discuss the latest gene editing technologies and discoveries. This Meeting Review describes the scientific event and highlights the main outcomes of both the conferences and the practical sessions. The symposium was a real success and thrives to educate, train and network international and young scientists in the field of gene editing and gene engineering.

## Teaser

**Summary:** This Meeting Review describes the African conference and workshop on CRISPR technology and highlights the main outcomes of both the conferences and the practical sessions. The symposium was a real success, helping to educate, train and network international and young scientists in the field of gene editing and gene engineering.

## Introduction

Clustered regularly interspaced short palindromic repeats (CRISPR) is a bacterial defence system that can cut DNA, first discovered in the DNA sequences of Escherichia coli bacteria (Duckett and Koop, 1977). To bind and cut DNA, CRISPR works with a nuclease called Cas and a guide RNA (gRNA) sequence that directs Cas to its target. Since the discovery of its ability to cut DNA, this mechanism has been used in laboratories for basic molecular research and has rapidly developed into a tool for editing genomes (Ran et al., 2013). Nobel laureates Emmanuelle Charpentier and Jennifer Doudna first adapted CRISPR-Cas9 as a gene-editing tool over 10 years ago (Jinek et al., 2012), and the technology has been rapidly developed and used for many different purposes in a wide range of organisms, including humans. We can name RNA editing, base and prime editing, live imaging and diagnostics, and some treatment clinical trials are already underway (Huang et al., 2022; Pisciotta and Pareyson, 2023; Zha et al., 2023).

## Why an African conference on CRISPR technology?

This astonishing growth in the use of CRISPR technology has also had its downside, especially in universities and laboratories facing budget cuts and infrastructure difficulties, which is essentially the African research environment.

In fact, after 15 years spent in Europe for my PhD project and several postdoctoral and research positions, I dropped my bags in my home country Tunisia to join the Faculty of Science and the Faculty of Medicine of Tunis as an assistant professor in 2022. That same year, I was invited to a national, North African and international symposium on life sciences, and while discussing their projects with students and researchers, I noticed that CRISPR technology was considered science fiction. CRISPR has been linked to clinical trials, rare diseases, high technology and high-cost labs. The story of the gene-edited twins in China was also a source of confusion (Raposo, 2019). A conference and workshop to introduce CRISPR technology from scratch and its utility in low-budget labs was then a must. It is something I had to do.

## What was the main theme of the colloquium?

In order to organise such an exciting scientific event, I and the committee I had formed chose the Institut Pasteur de Tunis (IPT) (http://www.pasteur.tn/) as the main venue. The Institute carries out epidemiological and clinical studies, biomedical research and research on human and animal health. The main theme of the conference was the low budget model. The speakers were introduced in crescendo according to their model.

The conference started Monday 12th June 2023 with Antony Adamson from Manchester University with a talk named ‘The 4D's of CRISPR: Design, Develop, Deliver and Detect’ to introduce CRISPR technology. Then Janine Scholefield from The Council for Scientific and Industrial Research in South Africa took the lead and gave a talk to contextualise the technology in Africa named ‘Using genome engineering to model disease in Africa: lessons learnt from a resource limited setting’. Pia Johansson from Lund University, Sweden ended the session with a talk named ‘The non-cutting, cutting-edge gene editing technology: CRISPRa and CRISPRi’.

The second day was dedicated to ‘Petri dish’ micro-organisms and it started with Sadri Znaidi from IPT with a talk named ‘CRISPR-Cas9 applications in eukaryotic pathogens: *Candida albicans* model’ followed by Thouraya Boussoffara, also from IPT and talking about ‘CRISPR-Cas9 Applications in Eukaryotic Pathogens: Leishmania major model’. Nicolas Talarek from the Institute of Molecular Genetics of Montpellier, France gave a conference about ‘CRISPR-Cas9 in *Saccharomyces cerevisiae* model’ and Wided Kelmemi [Merck KGaA, Darmstadt, Germany] ended the session with a symposium about the collaborative effort from MERCK group to enhance gene-editing technology in African labs.

Day 3 was dedicated to small models, namely zebrafish and Drosophila. Anabela Bensimon-Brito from the Marseille Medical Genetics Centre, France, started with a talk entitled ‘Generating genetic tools in zebrafish – models for tissue development, regeneration and disease’, followed by Maria Dolores Martín-Bermudo from the Centro Andaluz de Biología del Desarrollo, Spain, and Isabel Palacios from Queen Mary University of London, UK, with talks entitled ‘Using CRISPR in flies to identify modulators of tumurogenesis’ and ‘CRISPR in flies and in human organoids’, respectively. The session ended with a presentation by Sophien Kamoun and James Canham from The Sainsbury Laboratory and the GetGenome Foundation, UK, who introduced GetGenome, a project that aims to help scientists from the so-called Third World gain equitable access to genomic technologies and genomics-related training and education.

Day 4 focused on the diagnosis and treatment of human diseases. It started with a talk by Insaf bel Hadj Ali from IPT entitled ‘CRISPR Cas system for infectious disease diagnosis’, followed by Misaki Wyangera from Makere University, Uganda and a conference entitled ‘Gene editing to fight against Human Immunodeficiency Virus’. The session concluded with Professor Keith R. Jerome from the University of Washington & Fred Hutchinson Cancer Research Centre, USA, presenting ‘Gene editing to fight against infectious diseases’.

Day 5 was also a high-quality conference, starting with a talk by Laurie Menger from the Institute of Cancer Research Gustave Roussy, France, entitled ‘CRISPR in T-cells as solution to modulate cancer immunotherapy’ and followed by a presentation by David Savage from the University of California, Berkley, CA, USA, entitled ‘High-throughput approaches in to engineering CRISPR proteins’.

**Fig. 1.**
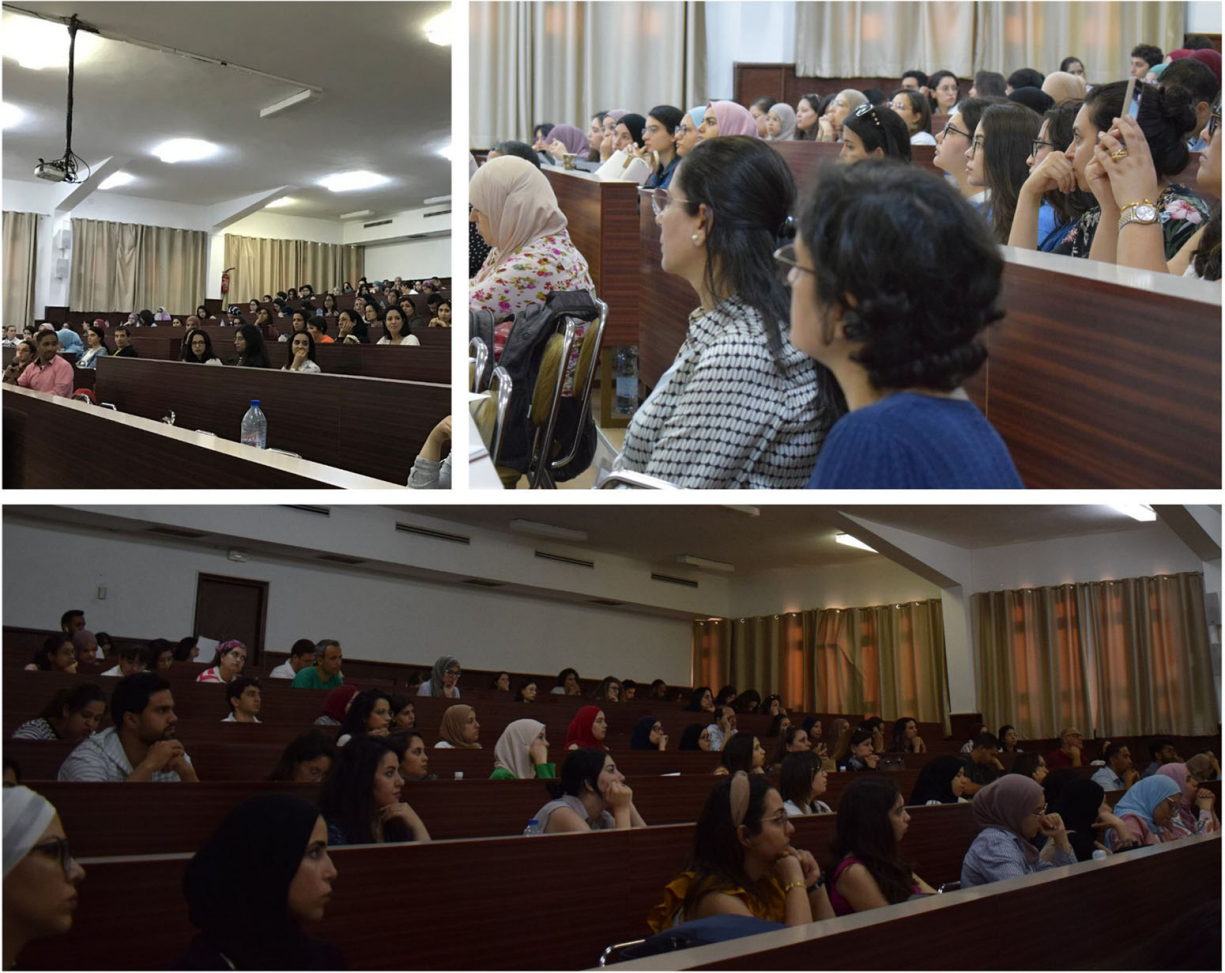
Daily attendance overview for conferences.

**Fig. 2.**
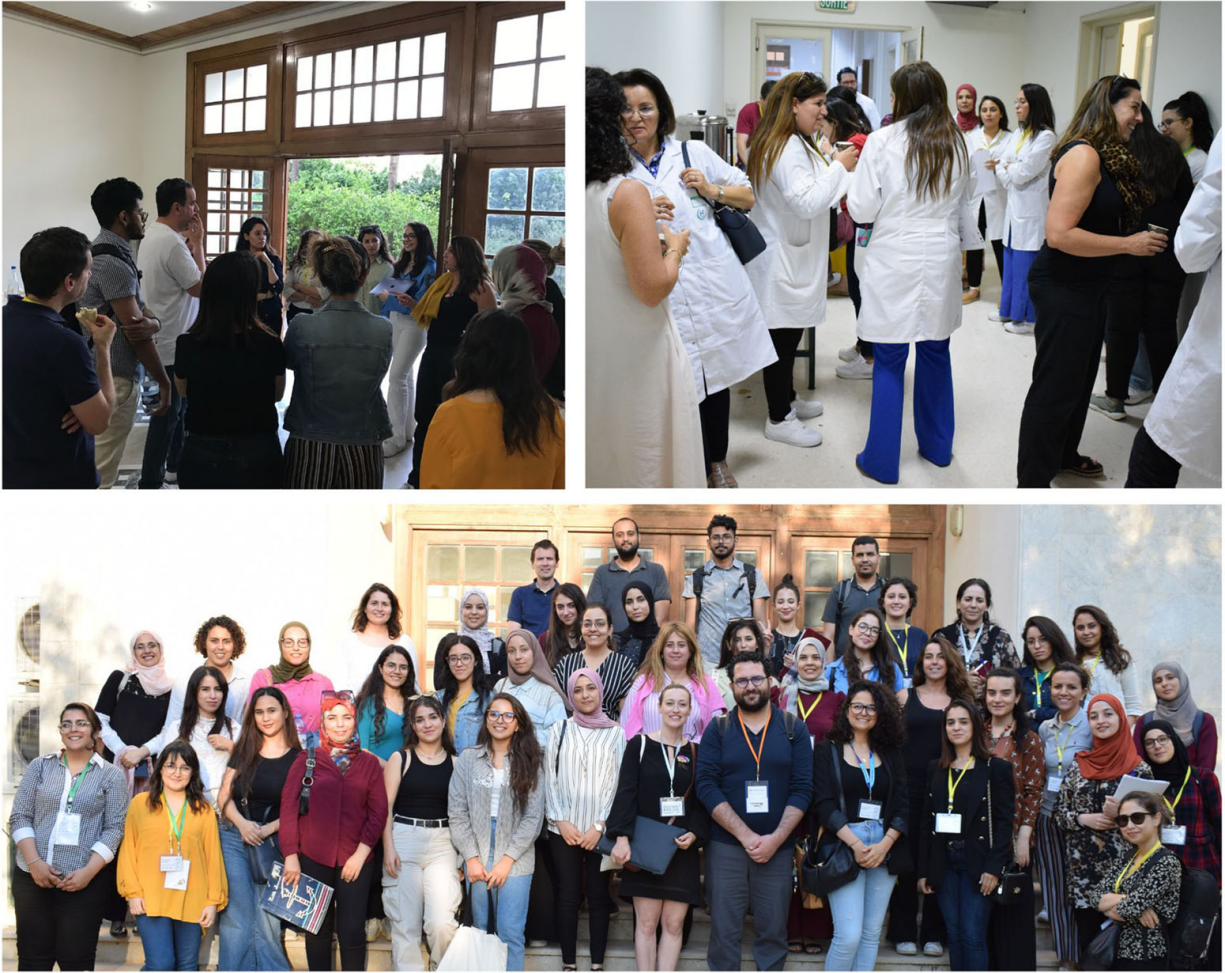
Informal networking sessions.

The 5-day seminar was Open Access and no fees were charged to participants. We had almost 350 pre-registrations and around 150 participants in the amphitheatre each day (Fig. 1). Most of the conferences were also recorded and available on YouTube (https://www.youtube.com/playlist?list=PLXohrS5wWCq_SLNNLnIhQuXmXMLQO3C85). Participants from Tunisia and further afield including from Algeria, Morocco, Mauritania, Iran, France and Italy. To encourage collaboration with the students, researchers and speakers on site, we organised an informal networking session each day with snacks, drinks and, of course, brainstorming and innovative ideas (Fig. 2).

## Workshop and practical sessions

**Fig. 3.**
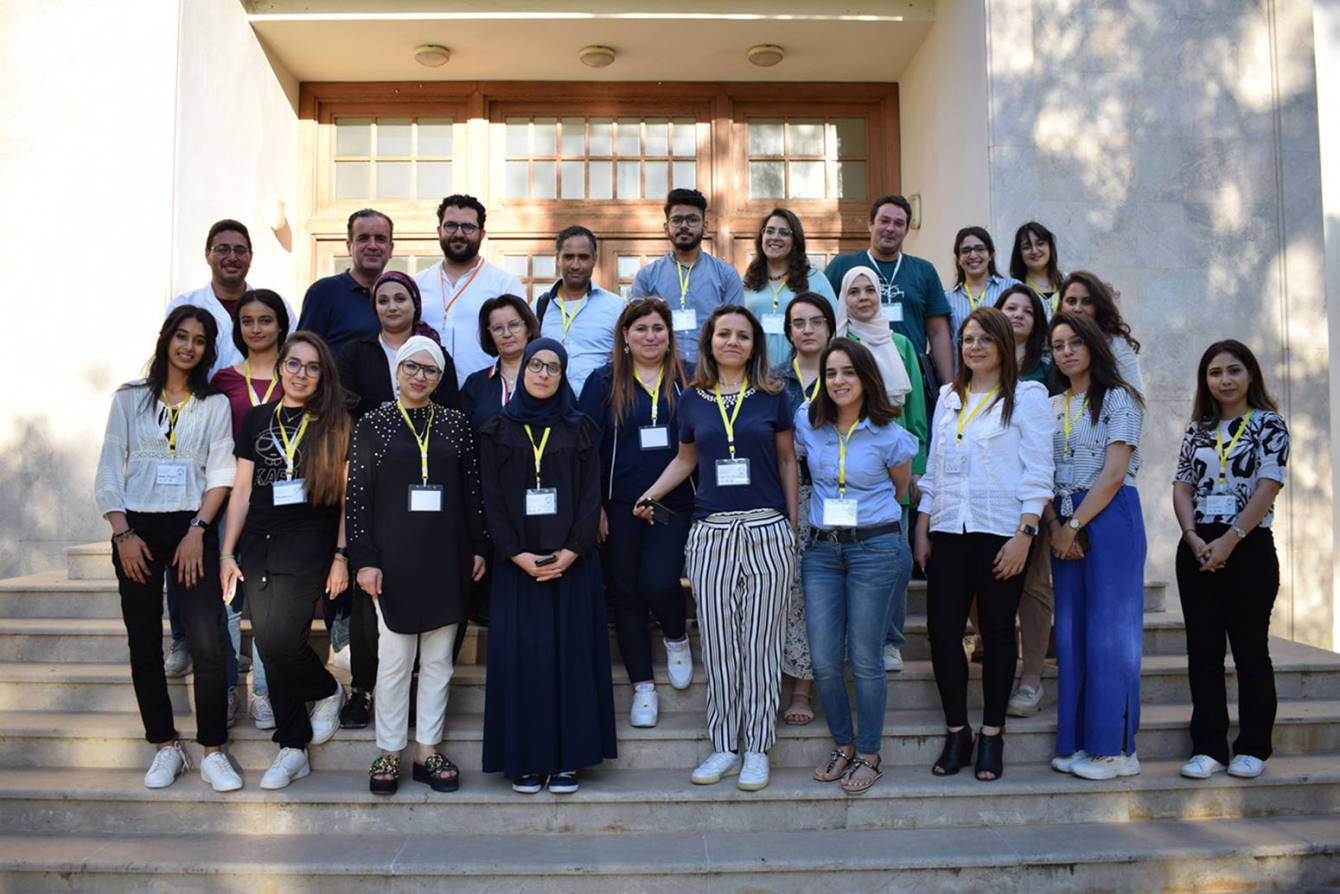
Selected candidates for the practical sessions.

In addition to the daily Open Access conference, we also organised practical sessions for selected candidates (postdocs and PhD students). We received more than 80 applications and due to the limited number of places we selected 17 candidates from Tunisia, Morocco, France and Italy (Fig. 3). The selection was based on the need for CRISPR technology in the candidate's research project and the biological model.

**Fig. 4.**
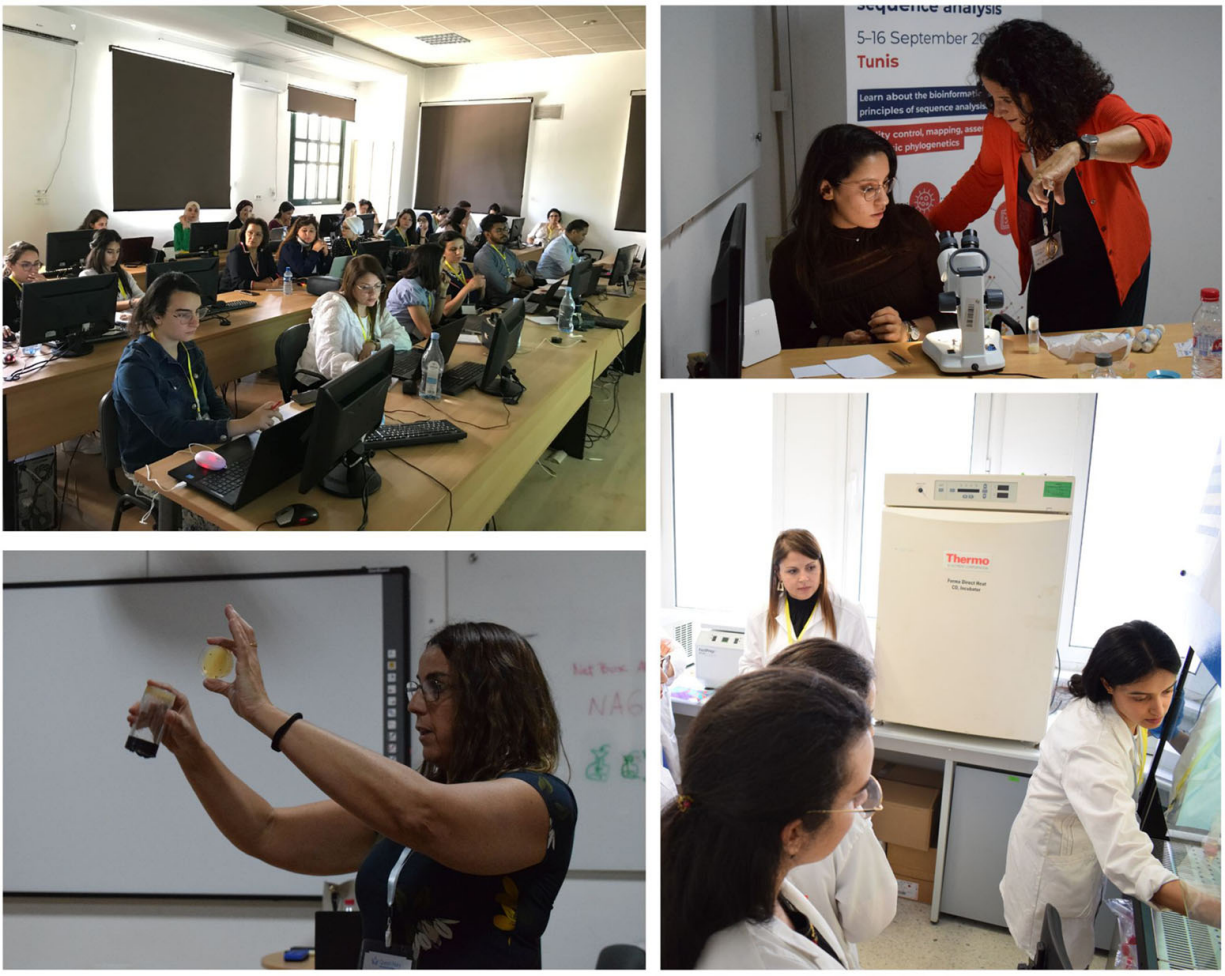
An overview of the practical sessions.

**Fig. 5.**
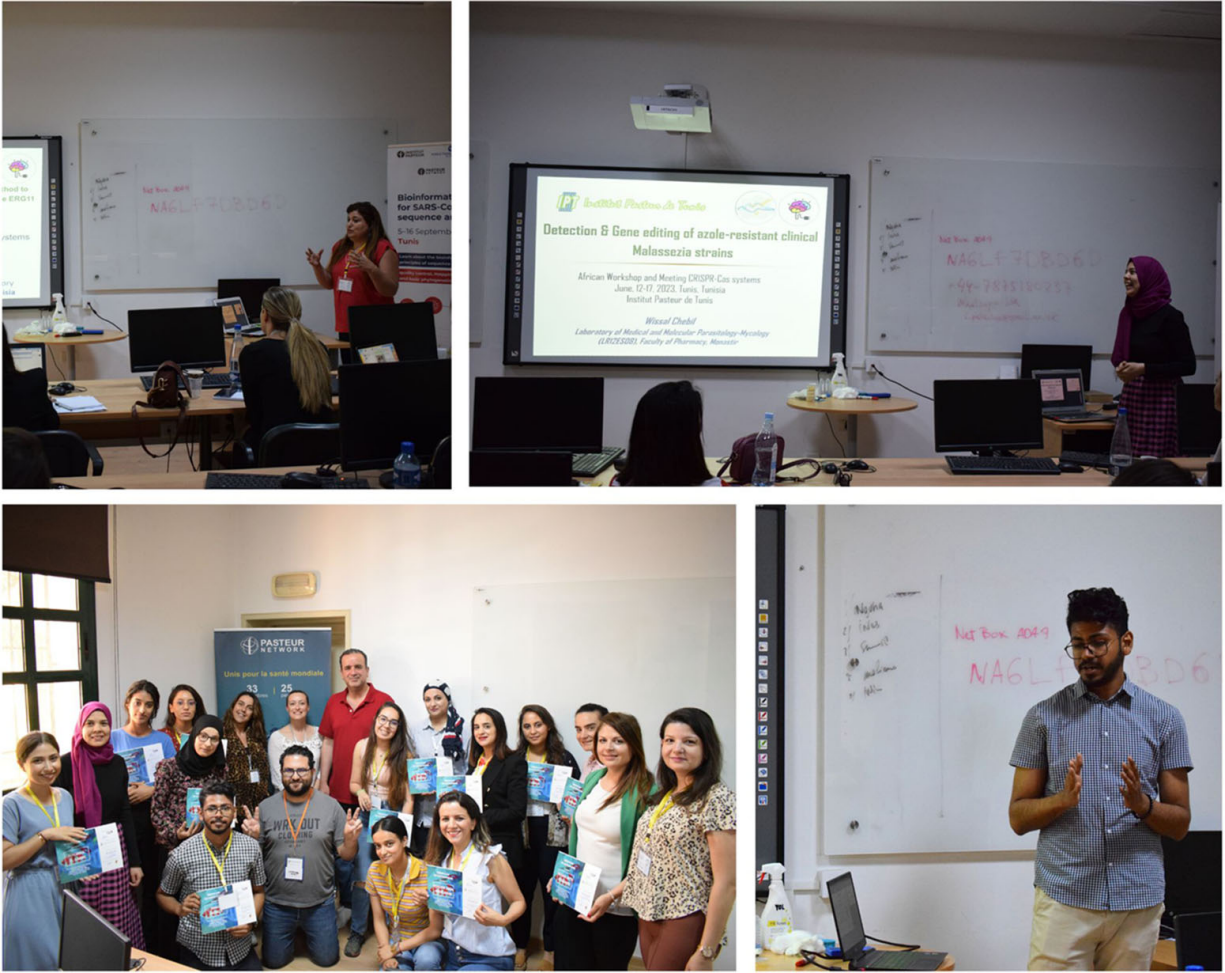
**Presentation of candidates**’ **projects and certificates awarded.**

The practical sessions were structured as follows, we began by introducing the participants to the notion of experimental design, the choice of free and open software, such as the Eukaryotic Pathogen CRISPR guide RNA/DNA Design Tool (EuPaGDT) (http://grna.ctegd.uga.edu/), the Eukaryotic Pathogen, Vector and Host Informatics Resource (VEuPathDB) (https://tritrypdb.org/tritrypdb/app/) and the well-known web tool for the selection of target sites for CRISPR/Cas9, CRISPR/Cpf1, CRISPR/Cas13 or NICKASE/TALEN-directed mutagenesis CHOPCHOP (https://chopchop.cbu.uib.no/), among others. The sessions then moved to the bench for 4 days to carry out the planned experiments on the yeast *Saccharomyces cerevisiae*, the fungus *Candida albicans* and the fly *Drosophila melanogaster* (Fig. 4). The practical session ended on day 6 with a presentation of the participants’ projects and a discussion with the trainers and speakers for advice and future networking (Fig. 5).

## Take-home message

Essentially, CRISPR remains a molecular biology tool that can be used in any living system, and because of its robustness it has become a key tool in the diagnosis and treatment of several diseases. However, for research purposes, especially in low-budget labs, scientists should not forget the basics: CRISPR is a molecular biology tool, and that means:

1. Flexibility: CRISPR can be used in a wide range of organisms, from bacteria and plants to animals and humans.
2. Precision: CRISPR permits highly targeted and precise modifications to the DNA and de facto minimizes the risk of off-target effects compared to older gene-editing methods.
3. Speed and efficiency: The technology is relatively fast and efficient compared to traditional gene-editing methods. It can produce genetic modifications in a matter of weeks, whereas older techniques often took months or even years.
4. Ease of use: The design and implementation of CRISPR experiments is becoming increasingly democratised with the advent of open access tools.

This feature will undoubtedly reduce the cost of experimentation and enable African laboratories and researchers to carry out high-quality genetic research and engineering. The quality will attract effective collaborations, and collaboration will lead to improved research and education to advance African countries.
